# Challenges and enablers for implementation of WHO ‘Best buys’ interventions targeting risk factors of diabetes and hypertension in South Africa: a mixed methods study

**DOI:** 10.11604/pamj.2022.43.215.31547

**Published:** 2022-12-30

**Authors:** Jeannine Uwimana Nicol, Lynn Hendricks, Taryn Young

**Affiliations:** 1 Centre for Evidence-based Healthcare, Division of Epidemiology and Biostatistics, Department of Global Health, Faculty of Medicine and Health Sciences, Stellenbosch University, Cape Town, South Africa,; 2 School of Public Health, College of Medicine and Health Sciences, University of Rwanda, Kigali, Rwanda

**Keywords:** Challenges, opportunities, population level interventions, WHO best buys, risk factors, diabetes, hypertension, non-communicable diseases, mixed methods, South Africa

## Abstract

**Introduction:**

World Health Organization (WHO) recommends the implementation of ‘Best buys’, cost-effective interventions that address risk factors of non-communicable diseases (NCDs). However, country responses to the implementation of these have been slow and undocumented. The aim of this study was to identify and understand challenges and enablers for the implementation of WHO ‘Best buys’ for risk factors of diabetes and hypertension in South Africa (SA).

**Methods:**

a mixed methods research with a sequential dominant status design was used starting with a document review to take stock of WHO ‘Best buys’ in policy in SA. A qualitative study using key informant interviews was then done to identify and understand challenges and enablers for implementation. A triangulation approach for the analysis of both document review and qualitative data was used.

**Results:**

SA has made good progress in including the WHO ‘Best buys’ in the policy. However, several challenges hamper the successful implementation. Most challenges were related to upstream policy implementation processes such as competing interests of actors, lack of balance between economic vs health gains, and lack of funding. Enabling factors included multi-sectoral engagement and collaboration; community ownership and empowerment; building partnerships for co-creation of enabling environments; leveraging on the existing infrastructure of other health programs; contextualization of policies and programs; and political will and leadership.

**Conclusion:**

SA has made good progress in including WHO ‘Best buys’ targeting risk factors of diabetes and hypertension in policy, however, various contextual barriers influence effective implementation. Hence, there is a need to leverage enabling factors to foster the implementation of WHO ‘Best buys’ interventions targeting risk factors of diabetes and hypertension in South Africa.

## Introduction

Non-communicable diseases (NCDs) claim about 41 million deaths each year [1] with hypertension and diabetes being major contributors to morbidity and mortality worldwide [2]. The number of people living with diabetes in Sub-Saharan Africa (SSA) is now estimated at 12.1 million people, and this number is expected to rise rapidly, reaching 23.9 million by 2030 [2]. In South Africa (SA), both hypertension and diabetes, are major contributors to morbidity and mortality [3]. In 2013, the SA government committed to reduce by at least 25% the relative premature mortality (under 60 years of age) from NCDs by 2020 through its strategic plan for NCDs [4]. Both population-level interventions addressing modifiable risk factors and integrated health service delivery platforms can contribute to reduction of morbidity and mortality [5]. Some of the modifiable risk factors for cardiovascular disease (CVD) and diabetes include poor diet, insufficient physical activity, and excess body weight. Thus, the risk factor distributions at the population-level can potentially be changed by changing the lifestyle of people, the environments where they live or work, that mitigate the lack of physical activity, smoking, and diet.

The United Nation's sustainable development goals (SDGs) included the NCDs with a target of reducing premature mortality by a third in 2030 [6]. But the current trend in decline of global rate of premature deaths from NCDs is not enough to meet this target [7] amidst of existing evidence based interventions such as the WHO ‘Best buys’, which have proven to contribute to the reduction of premature mortality from NCDs if they are well implemented [1]. WHO ‘Best buys’ endorsed in 2017 comprised of 16 practical and cost-effective interventions that work and can be delivered at the primary health level. Critically, these interventions put the emphasis on promoting health and preventing disease, and include interventions such as increasing tobacco taxes; restricting alcohol advertising; reformulating food products with less salt, sugar and fat; vaccinating girls against cervical cancer; treating hypertension and diabetes [1].

Country responses to implementation of WHO ‘Best buys’ have been low and uneven [4,8]. This could be due to various factors including contextual and policy agenda setting process. Breda and colleagues argue that most of the countries particularly in low- and middle-income countries (LMICs) do not have the capacity to implement these global policies and interventions at scale [8]. In addition, contextual factors that have hampered implementation of global policies or interventions such as the WHO ‘Best buys’ include lack of proper adaption of these global policies to fit the specific social, cultural, economic, political, legal, institutional settings and physical environments in which their being implemented [8,9]. Hence, it's important when global policies and, or interventions are implemented at country level to have engagement of stakeholders from all levels of policy making cycle, prevention and management of diseases form the onset of the planning stages, and across all stages of implementation process, to ensure appropriate and effective implementation [8,10-12]. This is paramount for the implementation of health interventions, in particular, given the multi-dimensional determinants of chronic conditions requiring interventions beyond health.

Beside lack of multi-sectoral collaboration as a barrier for effective implementation, other factors such conflicting priorities or agenda between different sectors of the same government often leads to disagreements on acceptable interventions and policies [8,13]. Lack of dedicated resources and investment to support coordinated implementation, monitoring and evaluation of NCDs interventions were highlighted in studies conducted in Zambia, Bangladesh and Iran. This study aimed to determine challenges and enablers for implementation of WHO ‘Best buys’ for risk factors of diabetes and hypertension in SA.

## Methods

**Study design and setting:** this is a fully mixed sequential dominant status design [14], whereby a document review was conducted first, followed by a qualitative study. Mixed methods research approach [15,16] was used in order to gain a better understanding on the implementation of the existing policies and programs in South Africa in line with the WHO ‘Best buys’ [1] to address risk factors for diabetes and hypertension, and challenges and opportunities for its implementation. The WHO Global Strategy for Diet, Physical Activity, and Health (DPAS) was used to conceptualize and visualize the kinds of interventions of interest [17]. The DPAS strategy organizes intervention into three types that focus on supporting policies, programs, and the environment programs. These three categories of interventions were aligned with the WHO ‘Best buys’ to inform data collection tools and analyses.

The document review focused on identifying and reviewing the existing policy documents (policies, acts, regulations, guidelines, reports, etc.) and programs that addresses NCDs in SA with emphasis on policies and programs targeting risk factors of diabetes and hypertension. A review of relevant reports, journal articles, or policy documents was conducted from November 2019- March 2020. Government reports that contain information regarding the planning, implementation and evaluation of population-level interventions targeting diabetes and hypertension were considered. Various databases were used to run the search and there was no restriction of date and language applied to the search.

The document review informed the mapping of stakeholders to be involved in the key informant interviews (KIIs) by identifying experts in supportive policies, programs and enabling environments for NCD prevention in South Africa. The KIIs were conducted to get a deep understanding of challenges and enabling factors for implementation of population WHO ‘Best buys’ in SA.

**Data collection:** for the document review, various databases were searched and these included Pubmed, Ebscohost, Google scholar and Scopus and Cochrane database. Search terms used included diabetes /hypertension/ physical activity/ nutrition/ alcohol consumption/ tobacco smoking/ programs/ interventions/ South Africa. There was no restriction of date and language applied to the search. An excel spreadsheet was designed to extract relevant information on the type of intervention, coverage, target audience, and if there have been any process or impact evaluation. A total of 13 participants who were representative of key stakeholders at national and provincial government departments and agencies involved in NCDs related programs as well as representatives of NGOs and food industry; and representatives of academic and research institutions were purposively selected and interviewed. These interviews took place in April- June 2021 using Microsoft Teams or Zoom due to the COVID-19 restrictions. Interviews were transcribed verbatim, captured, and coded using NVivo [18].

**Data analysis and management:** a triangulation approach for analysis of both document review and qualitative data was used. The data were compared and contrasted in order to achieve as rich picture of the situation as possible and to increase credibility the study. The data from the document review were analysed narratively. While the qualitative data were analysed thematically using the five stages of the framework approach as described by Pope and Mays [19]. The framework was chosen as it enables the researcher not only to focus on the pre-defined set of questions, but also to consider other themes emerging from the analysis.

An analytical grid of key themes was developed in lined with the study aims and familiarisation with the first few transcripts, and then applied to the rest of the transcripts. The WHO DPAS [17] strategy was used as thematic framework for analysis which organizes intervention into three types that focus on supporting policies, programs and the environment programs. These thematic areas were applied to the set of interventions outlined in the WHO ‘Best buys’ [1] by exploring their implementation in South Africa.

A constant comparison process was used during analysis, where all relevant data for each category of participants were identified, examined and compared with the rest of the data, to identify dominant themes occurring in all groups. The non-dominant themes were also grouped separately and compared across categories of participants to identify similarities and inconsistencies. Codes and themes were discussed between two authors (Jeannine Uwimana Nicol and Lynn Hendricks) until consensus was reached. To ensure trustworthiness and credibility of qualitative data procedures described by Mays and Pope [19], triangulation of data was observed to enhance trustworthiness of qualitative data [20].

**Funding:** the review work was supported by the funding from the Collaboration for Evidence-based Healthcare and Public Health in Africa (CEBHA+) project which is funded by the German Federal Ministry of Education and Research (BMBF) as part of the Research Networks for Health Innovation in sub-Saharan Africa Funding Initiative. The funder doesn´t have any role in the review process.

**Ethics approval:** ethics approval for this study was gained from The Health Research Ethics Committee at Stellenbosch University (N19/01/001). Consent for participation was provided by participants through a signed consent form.

## Results

Twenty-eight policies, legislations, strategic plans and regulations were identified - 8 policies on tobacco use (smoking); 7 policies on harmful consumption of alcohol; 8 policies on unhealthy diet and 5 policies of physical inactivity (PA). Thirteen supportive programs were identified of which 6 targeted unhealthy diet, 3 tobacco smoking and 4 targeted PA. These reviewed documents indicate that all the WHO ‘Best buys’ recommended interventions have been enacted and included in policy in SA (Table 1).

**Table 1:** implementation of WHO ‘Best buys’ interventions in South Africa to date

Risk factors	Interventions	Progress

Tobacco use	Tobacco price increases (tax increases)	xxx
	Smoke -free-indoor workplace and public places	xxx
	Health information and warnings	xxx
	Bans on tobacco advertising, promotion and sponsorship	x
Harmful alcohol use	Alcohol price increase (tax increases)	xxx
	Restricted access to retailed alcohol (purchase age, restricting locations and hours, government monopoly)	x
	Bans on alcohol advertising and sponsorship	xxx
Unhealthy diet and physical inactivity	Food reformulation to reduce salt content (reduced salt intake in food)	xxx
	Food reformulation to exclude saturated and trans fats	xxx
	Fiscal measures that increase the price of unhealthy foods or decrease the price of healthy foods	xxx
	Food labelling restrictions on marketing of unhealthy foods and beverages	x
	Mass-media campaigns to reduce salt consumption	xx
	Public awareness through mass media on diet and physical activity	xx
	Modification of built environment to promote physical activity	xx
**Specific intervention on diseases**		
Cardiovascular diseases and diabetes	Counselling and multi-drug therapy for people with a high risk of developing heart attacks and strokes (including those with established CVD)	xxx
	Treatment of heart attacks with aspirin	Xxx

Fully implemented xxx, Partially implemented xx, In progress/Inadequate x

**Figure 1:**
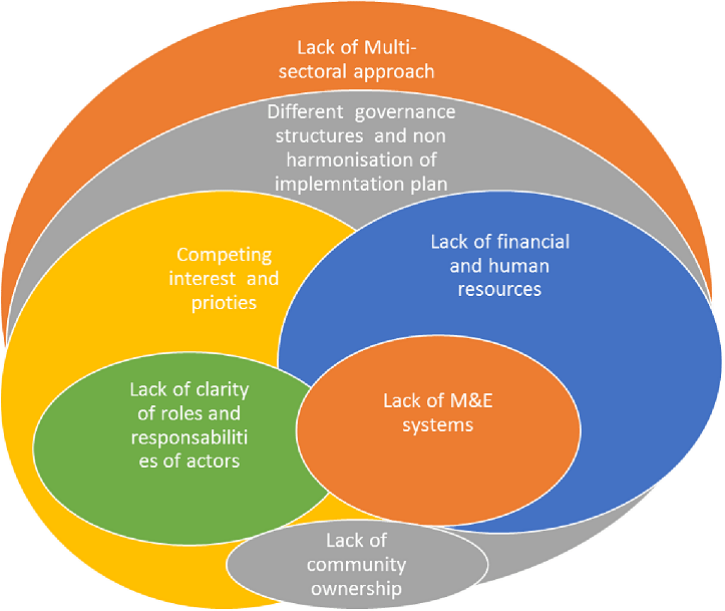
challenges for implementation of WHO ‘Best buys’ interventions

**Challenges for implementation of WHO ‘Best buys’:** governmental and industry stakeholders agreed that national policy implementation varied across provinces in South Africa, with some having more organized provincial structures and doing better than others. The predominant themes identified as challenges to the implementation of supportive policies and programs are summarised in Figure 1 and Table 2 provides challenges with illustrative quotes. Through the KIIs, the predominant themes identified as challenges to implementation of WHO ‘Best buys’ included among others lack of multi-sectoral approach; different governance structures and non-uniformity of provincial implementation plan; competing interests and priorities among stakeholders; lack of financial and human resources; lack of monitoring and evaluation systems; lack of community ownership; and inadequate communication strategy to promote behaviour change.

**Table 2:** summary of challenges for implementation of WHO ‘Best buys’ interventions with quotes

**Competing interests and priorities**	“Some provinces are able to do it better than others it might have to do with resources, it might have to do with priorities that are set for the different provinces. So, while these are national policies and should be implemented nationally it is not always clear whether that is the case and to what extent provincial health departments are prioritizing aspects of that. It might also just be because they have different health issues that they are prioritizing …” “I don't think people or policy makers, or practitioners are in denial, it's just they're so over-whelmed with so many competing health priorities that sometimes focus is not enough on a specific condition”
**Different governance structure and non-harmonisation of implementation plan**	“What we find at provincial level, is that different provinces have got different set ups, you find provinces that are well organised in terms of the structures that they have that are well integrated, there is various role players that are participating in those kinds of structures, but you go to the next province there isn't so much in terms of such extensive structures.”
**Lack of clear roles and responsibilities**	“I think roles and responsibilities are really crucial because it helps to clarify for people who should be doing what because when it comes to implementing there can be a lot of passing of the buck because people are busy”
**Balancing behavior change to reduce the risk of NCDs versus determinants of health**	“People are more worried about not having a house, not having food in their household, now you come with a small thing like don't eat salt, don't eat sugar, it's not important, when they have so many other things that they do not have. In fact the few people you speak to will tell you that if they don't have food in their house, why would they even bother about the amount of salt that they're using, you know, so there are a lot of things that for us to be able to change some of the behaviours”
**Lack of communication strategy promoting behaviour change**	“Yes, for instance the salt reduction, whilst you say for industry to reduce salt in their products, the behaviour of people has not changed much because they still add salt and when it comes to communication, communication is very costly, we do it but I don't think we do enough, so the challenge with some of these regulations, it's more about behaviour change which…it's not an easy…”
**Lack of community ownership**	It's the people. This should be a household community program. Do people want this program? And you can buy seeds for people, you can give them a “plantjies” or seedlings and give them too gardening tools to do home gardening. If they don't want it, they will not do it. So, it should be people who should see the value and want to do it. If it is still led by government, it will not succeed. The moment you walk out, it collapses.”
**Unethical sponsorship**	“We passed a resolution at our last conference where we said that government should not be accepting money from those type of donors [unhealthy foods and beverages] and of course the Minister of Sports at that time said well then how do we fund or how do we then allow programs to continue? Sports industries will have to come to an end. So, these are the issues. It all runs around finance, around money, how do they generate funding and money for themselves and what are the other opportunities that they can get to be able to sponsor and to run these programs”

***Lack of financial and human resources:*** lack funding was identified as an important impediment for both policy formulation and policy implementation. Limited funding allocated to provinces and NGOs at large was identified as the main hindrance to effective implementation of population-based interventions. Some stakeholders working in the research industry expressed that lack of funding to do research relevant to the local health needs within the African context to inform policy has been a challenge.

***Competing interests and priorities among stakeholders:*** conflict of interest between the national trade and investment policy vis a vis the national health policy whereby trade, and foreign direct investment have a tendency of promoting the influx of large amounts of processed foods and sugary beverages, alcohol as well as smoking has been perceived by participants as among the challenges that hinder the implementation of both supportive policies and programs targeting diabetes and hypertension in particular the ones that target unhealthy diet, smoking and alcohol consumption. Also, stakeholders perceived that prioritization of policies differed across government departments and between national and provincial government. Sometimes prioritization of policies and programs to be implemented depends on the availability of resources which explains why some provinces have enabling environments to promote physical activities (i.e. cycling lane, outdoor gyms, parks, etc) and some do not have. Thus affect the implementation of supportive policies nationwide. When reflecting on the implementation of the Integrate Food and Nutrition Security Strategy, participants expressed that while the strategy was developed in consultation with various national government departments and agencies, the implementation plan could not be fully funded due to competing budget interests. *“Well, the implementation … okay, you know, we have drafted that [Integrate Food and Nutrition Security Strategy] implementation plan. When we finalized the plan we wanted it approved by cabinet. And they said no, no, where did you think, we are going to get all this money? And they said go back to your respective departments and reprioritise.”* (SH-AAD1)

***Lack of monitoring evaluation (M and E) system:*** lack of proper of M and E system for NCDs program has been identified as a challenge for effective policy and program implementation. Also, non-integration of NCDs key data elements within existing electronic M and E system (i.e DHIS, Tier.net) for other health program such as HIV has been problematic in measuring the effect of some policy interventions. *“TB and HIV have the Tear.net system and we have requested multiple times to just put in two additional fields to collect data for hypertension and diabetes, but you know there are so many patients who have co-morbidities and it would make such a difference because Tear.net generates their own reports. We would be able to get so much information based on the Tear.net system, unfortunately we were turned down so many times and even now with the revised Tear.net system, we weren't even offered that opportunity to include those two fields”* (SH-ND2)

***Different governance structures and non-uniformity of implementation plan:*** governmental and industry stakeholders agreed that national policy implementation varied across provinces in SA, with some having more organized provincial structures and doing better than others. One of the policy makers argue that *“What we find at provincial level, is that different provinces have got different set ups, you find provinces that are well organised in terms of the structures, they are well integrated, there are various role players that are participating in those kinds of structures. But you go to the next province there isn't so much in terms of such extensive structures.”*

***Lack of clear roles and responsibilities of actors:*** there was a perceived connection made by participants between policy implementation success and coordinating mechanisms with provision of clear guidelines on roles and responsibilities. Lack of such clear directives for implementation of these population-level interventions impact on the success of these interventions and at times could lead to tension among actors/implementers. *“One of the challenges in the implementation of the NCD strategy was lack of responsibility for by the NCD cluster overseeing the implementation. There was no time at that stage to develop an agreement on how the programmes would work together to contribute to the set targets. Also, the success of that strategy would have been largely dependent on a very strong coordinating mechanism both within the programmes within the department, but also between the department and other sectors outside the department”* (SH-ND4).

***Balancing behavior change to reduce the risk of NCDs versus determinants of health:*** behaviour change at individual and community level is required to reduce the risk of NCDs, however, this is especially challenging. For example, the Salt Reduction Policy is one of the policies related to healthy diet that has been identified as a policy that engaged extensively the food industry in its formulation. But the policy's implementation process was largely dependent on a communication strategy promoting behaviour change with mass education and campaigns activities at community level. The implementation of the policy has been problematic due to the heavy cost involved in executing the communication strategy/programs. Also, another complexity in promoting behaviour change is the existence of determinants of health such as poverty, access to basic services such as housing that makes a difficult choice for people to opt for behaviour change such as healthy diet.

**Enablers for implementing WHO ‘Best buys’:** the enabling factors for effective implementation of WHO ‘Best buys’ most predominantly identified by participants across all categories included multi-sectoral engagement and collaboration; community ownership and empowerment; building partnerships for co-creation of enabling environments; leveraging on existing infrastructure of other health programs; contextualisation of policies and programs; community driven activism; balancing economic versus health gains; and political will and leadership. Table 3 summarises enabling factors for implementation of WHO ‘Best buys’ with illustrative quotes.

**Table 3:** enabling factors implementation of WHO ‘Best buys’ interventions with quotes

**Community ownership and empowerment**	“If you are engaged with different communities and share with the communities' evidence about dangers of alcohol consumption…you are able to get by and work with us to implement policies especially awareness about dangers of alcohol misuse. We will strengthen communities to work together to fight against alcohol use.” “… If they are empowered to understand the dangers of alcohol misuse they are the ones who will strengthen the implementation of policy because they understand where the policy comes from and what is the output of the policy and the outcome”
**Community driven activism for NCDs**	“The NGOs are involved…we need them a lot to advocate to various stakeholders, so they do that a lot and because they also work with communities out there, they are able to mobilise communities and they bring in a lot of evidence as well which we might not have”
**Inter-sectoral partnerships and engagement**	“WOW! has partnered with various companies including the Heart and Stroke Foundation” to “develop healthy tuckshop guidelines…”and …Kubeka bicycles and gave 3000 bicycles to learners in the Paarl area to help them, not only to be more physically active, but also to live a healthy life>
**Leveraging on existing infrastructure of other health programs**	“Bringing it from the perspective of HIV and TB, which has got a platform already and happening, which you can just update to that platform and expand to NCDs. We have seen millions of people screened for HIV and we are missing also screening them for other conditions such as diabetes and hypertension that are equally important.”
**Building partnerships for co-creation of enabling environments**	“In terms of physical activity, we started WOW! groups, you might have heard of, they offer a structured program to promote physical activity, not only in the home environment but in the community, and also at work- so it's different populations or different target groups…people meet, they walk, they network and it's just become quite a fun element”
**Creation of economic opportunities while promoting healthy living**	“People are producing food now in order for them to sell and be able to get an income from it. So now if there isn't clear markets that has been achieved for these indigenous foods, people will not be so eager to produce them because there will not be any economic gains from it, it will not form part of the rural economist that we are talking about. People will be more eager to be involved in producing crops that are more likely to get them their income that they're looking for”. “…high concentration of alcohol outlets contributes to alcohol misuse but on the other hand tavern owners will see it as an opportunity to have businesses where you make decision about maybe density of alcohol outlets it is not a straightforward thing you have to balance between economy as well as health.”
**Establish mechanism to promote compliance of industries**	“At private industries we need to make sure that, because people buy 90% of the food that people buy in South Africa comes from the food industry. So we have a role to play to make sure that we are spending more on nutritious food. And I think what consumers are increasingly asking for these foods anyway, so it is in our interest to make sure that we are providing nutritious food”
**Creating incentives that promote good performance**	“on an annual basis we have what we call the WOW annual award ceremony, that now will be in it's 5th year next year and this is what we look at, we look at the whole year's M&E data, quantitative and qualitative. We identify the top performing WOW groups and also the champions who've really performed outstandingly and it's not about the biggest loser, it's not about the group with the highest number of participants, it's about how innovative you are and despite all the odds, that you still achieve and you make change…”
**Political Will and leadership**	“There is that politically positive environment, even the district model approach…we should begin to see our political leaders supporting these initiatives …”

***Multi-sectoral engagement and collaboration:*** most participants expressed that one of the key drivers of a successful policy implementation was engagement and collaboration between government departments and with civil society, researchers, and communities. On an inter-departmental government level, engagement and working transversally was found to promote policy implementation as expressed by one of the participants: *“I think that is the reason why we are not making a huge, an impact, because really, if you look at it, it cannot be health alone.”*

***Community ownership and empowerment:*** for communities, a shared sense of ownership enable implementation. Working together with communities also included regular meetings and being responsive to their ongoing and changing needs.

***Building partnerships for co-creation of enabling environment:*** creating an enabling environment for making healthier choices includes building sports and recreational facilities as well as safe spaces for children to play. As much as there are indication of enabling environment intervention tailored to risk factors of diabetes and hypertension across the country, most of these interventions focuses on promotion of physical inactivity (i.e. Outdoor Gyms, cycling lanes, public parks) and their coverage vary according to each province, district, etc.

***Leveraging on existing infrastructure of other health programs:*** some participants were of the view that existing health programs such as TB and HIV provide an opportunity for integrating interventions geared to NCDs prevention such as diabetes and hypertension as expressed by one participant: *“Bringing it from the perspective of HIV and TB, which has got a platform already and happening, which you can just update to that platform and expand to NCDs. Because I could not understand they screen for HIV and not at the same time screen for hypertension and diabetes. It's just so simple during that same encounter, to actually take the blood pressure of people, you wouldn't add to the load.”*

***Civil society and community led activism:*** using civil society as an inter-mediatory to advocate policy implementation to stakeholders was found to be an enabler, as was reported with the Control for Tobacco Products and Electronic Delivery Systems Bill [21].

## Discussion

South Africa has a comprehensive spectrum of supportive policies and programs targeting risk factors for diabetes and hypertension and NCD prevention at large since the post-apartheid era [22]. These supportive policies and programs targeting risk factors for diabetes and hypertension were designed in line with the WHO ‘Best buys’ [1] with a national coverage (Table 1). This study triangulated findings from a desk review and qualitative data to identify challenges and enabling factors for implementation of WHO ‘Best buys’ related to risk factors of diabetes and hypertension in South Africa. Effective implementation of these interventions have been hindered by various factors such as lack of multi-sectoral approach; different governance structures and non-uniformity of provincial implementation plan; competing interests and priorities among stakeholders; lack of financial and human resources; lack of monitoring and evaluation (M and E) systems including non-integration of NCDs data elements into existing health information systems such as DHIS, HIV electronic; lack of community ownership; and inadequate communication strategy to promote behavior change.

These implementation challenges are not unique to South Africa. Lack of multi-sectorial approaches have also been reported by various researchers in LMICs [11,12,23,24]. Conflicts of priorities between government departments on regulations related the economic gains have been reported in other studies [22]. Malawi, Cameroun and Ghana reported how competing interests and priorities have hindered country progress on WHO FCTC and alcohol consumptions as well as unethical sponsoring of sports events by alcohol industry [11,23]. Furthermore, lack of M and E systems for NCDs in general has led to lack of baseline and representative data to assess the effectiveness of population level interventions and their impacts on NCD prevention and control have been reported in other countries [24,25]. Mukanu *et al*. [24] in their study on a review of NCDs policy in Zambia argue that there was no sufficient data to inform some of the activities stipulated in the Zambian NCD strategic plan such as salt reduction and increase in uptake of physical activity. Bourdeaux and colleagues argue that limited funds for NCDs prevention and control is due to the fact most LMICs countries get their funding from NGOs [21]. Moreover, lack of and limited funding for the implementation of NCDs strategic plans has a direct impact on establishment of M and E systems to assess the effectiveness and impact of the policies and programs emanating from these NCDs strategic plans.

Although the findings of this study highlight a number of challenges hindering the implementation of WHO ‘Best buys’, there are also enabling factors that could potential influence the effective implementation of these WHO ‘Best buys’. The enabling factors most predominantly identified by participants across all categories include multi-sectoral engagement and collaboration; community ownership and empowerment; building partnerships for co-creation of enabling environments; leveraging on existing infrastructure of other health programs; contextualisation of policies and programs; community driven activism; balancing economic versus health gains; and political will and leadership.

Juma *et al*. [11] in their study on NCDs prevention policy process in five African countries (South Africa, Malawi, Kenya, Cameroon and Nigeria) stress the importance of creating a coordination mechanism embedded in inter-sectorality/multi-sectoral approach to enable effective implementation of interventions targeting risk factors for NCDs including diabetes and hypertension. Haldane *et al*. [26] in their review argue that community engagement and empowerment is one the key factors for implementing strategies in health promotion and the prevention and control of chronic diseases such as diabetes and hypertension, and community participation contributes to favourable outcomes at the organizational, community and individual level.

There are few studies in South Africa and in LMICS in general that have been reporting on the implementation of WHO ‘Best buys’ interventions targeting risk factors of diabetes and hypertension, and other NCDs [27]. This study highlights challenges and enabling factors related to the implementation of these WHO ‘Best buys’ interventions in South Africa in order to inform local interventions needed for effective implementation. Additionally, the uniqueness of this study lies in the research methods approach used where by multiple sources of data were used and the engagement with various actors involved in policy making and program implementation to provide comprehensive reflection of contextual challenges affecting effective implementation of WHO ‘Best buys’ in South Africa. But also inform possible areas of future research through the identification of enabling factors of implementing these interventions. However, we recognised the limitations of this study which is the lack of national quantitative data to determine process outcomes and impact of these WHO ‘Best buys’ interventions on diabetes and hypertension; and NCDs in general.

## Conclusion

In light of the growing burden of diabetes and hypertension, and NCDs in South Africa, there is a need to tackle risk factors for diabetes and hypertension. SA has made a good progress in including WHO ‘Best buys’ targeting these risk factors in policy, however there are various contextual barriers influencing effective implementation of these interventions. It's important to leverage on enabling factors, such as bottom-up approach anchored in multi-sectoral approach, to foster policy implementation.

### What is known about this topic

- *The burden of NCDs in LMICs including South Africa is arising particularly the burden related to diabetes and hypertension which poses a threat to already overstretched health systems and human capital development*.
- *South Africa has a plethora of policies targeting risk factors of diabetes and hypertension, and has been slow in achieving the SDGs targets related to NCDs*.

### What this study adds

- *This study reports on the implementation of WHO ‘Best buys’ interventions targeting risk factors of diabetes and hypertension in South Africa*.
- *This study highlights challenges and enabling factors for implementation of WHO ‘Best buys’ interventions targeting risk factors of diabetes and hypertension in South Africa which will inform the revised national strategic plan for NCDs (2020-2025)*.
